# Supplementary material for: Prevalence of short interpregnancy interval and its associated factors among pregnant women in Debre Berhan town, Ethiopia
Source: PLoS One. 2021 Aug 2;16(8):e0255613. doi: 10.1371/journal.pone.0255613 (PMC8328324; doi:10.1371/journal.pone.0255613)
Supplement: S2 File — (DOCX) [file pone.0255613.s002.docx]

Annex 1- English Questionnaire

**INTRODUCTION**

- CIRCLE THE RESPONSE
- CONSIDER SKIP QUESTION

| **Part I- Socio- demographic and economic characteristics of respondents** | | | | |
| --- | --- | --- | --- | --- |
| **Questions** | **Response** | | **Remark** | |
| 1. What is your current age? | |  | |  |
| 1. Age at marriage | |  | |  |
| 1. What was your age in your first birth? | |  | |  |
| 1. What is your current marital status? | | A= Single  B= Married  C= Divorced  D= Widowed  E= other, specify------- | |  |
| 1. What is your Religion? | | A= Orthodox  B= Muslim  C= Protestant  D= Catholic  E= other (specify) | |  |
| 1. What is your Educational status | | A= no formal education  B= primary education  C= Secondary education  D= college and above | |  |
| 1. What is your current occupation? | | A= Self employed  B= Private employee  C= Government employee  D= House wife  E=Student  H= Other | |  |
| 1. What is your husband education | | A= no formal education  B= primary education  C= Secondary education  D= college and above | |  |
| 1. What is your Husbands Occupation | | A= unemployed  B= Self employed  C= Private employee  D= Government employee  E = Others | |  |
| 1. House hold monthly income in ETB | |  | |  |

| **II. Reproductive history** | | |
| --- | --- | --- |
| **Questions** | **Response** | **Remark** |
| 1. Did you used modern contraceptive methods before this pregnancy | A. yes  B. no  C. yes, but failed |  |
| 1. If q 11, is yes which type of contraceptive methods | A. Implanon  B. IUCD  C. oral pills  D. Depo Provera  E. barrier methods (condom, diaphragm…………)  F. other, specify------- | If Q 11, is **No** skip to Q 13 |
| 1. Have you attended ANC follow up in previous pregnancy? | A. yes  B. no |  |
| 1. If Q13, is yes how many visit (s) | A. one  B. two  C. three  D. Four and above | If Q 13 is **No** skip to Q 15 |
| 1. Duration of exclusive breast feeding in the previous birth |  |  |
| 1. The duration of breast feeding for the previous child |  |  |
| 1. Who will decide to use maternal health services (to use contraceptive, ANC visit, delivery and child birth, child immunization, breast feeding….)? | A. self  B. both wife and husband  C. only husband |  |
| 1. What is the source of information on health related issues? | A. mass media  B. health professionals  C. friends and relatives  D. no information |  |
| 1. How many children do you have before the current pregnancy? |  |  |
| 1. . Do you have a male child? | 1. yes 2. no |  |
| 1. Pregnancy intention | 1. intended 2. mistimed 3. unwanted 4. mistimed and unwanted |  |
| 1. What is the outcome of previous birth? | 1. Alive 2. Dead |  |
| 1. What was the sex of the previous birth? | A. female  B. male |  |
| 1. Mode of delivery in the previous pregnancy | 1. vaginal 2. cesarean section |  |
| 1. Before pregnancy, was your menstrual cycle regular? | 1. Yes 2. No |  |
| 1. Do you have experience infertility in your life? | A. yes  B. no |  |
| 1. The inter-pregnancy interval (between previous birth and current pregnancy, LNMP)    1. date of birth for the previous birth-----    2. LNMP------ |  |  |

Annex 2: Amharic questionnaire

መግቢያ

አቀራርቦ መዉለድ በእርጉዝ እናቶች አስመልክቶ የተዘጋጀ መጠይቅ

1. እድሜሽ ስንት ነው? -----
2. ስታገቢ እድሜሽ ስንት ነበር?
3. የመጀመሪያ ልጅሽን ስትወልጅ እድሜሽ ስንት ነበር?
4. አሁን ያለሽበት የትዳር ሁነታ

A= ያላገባ

B=ያገባ

C= የፈታች

D= ባል የሞተባት

E= ሌላ ካለ ተናገሪ-------

1. ሃይማኖትሽ ምንድ ነው?
2. ኦርቶዶክስ
3. ሙስሊም
4. ፕሮቴስታንት
5. ካቶሊክ
6. ሌሎች/ላ--------
7. የትምህር ደረጃሽ?

A= ምንም ያልተማረች

B= 1ኛ ደረጃ የተማረ

C= 2ኛ ደረጃ የተማረ

D= ኮሌጅ እና ከዚያ በላይ

1. ስራሽ ምንድነዉ?

A= በግል የተሰማራ

B= የግል መስሪያ ቤት

C= የመንግስት ሰራተኛ

D= የቤት እመቤት

E=ተማሪ

H= ሌላ ካለ ተናገሪ-------

1. የባልሽ የትምህርት ደረጃ?

A= ምንም ያልተማረ

B= 1ኛ ደረጃ የተማረ

C= 2ኛ ደረጃ የተማረ

D= ኮሌጅ እና ከዚያ በላይ

1. የባልሽ ስራ ምንድነዉ?

A= ያልተቀጠረ

B= በግል የተሰማራ

B= የግል መስሪያ ቤት

C= የመንግስት ሰራተኛ

E =ሌላ ካለ ተናገሪ-------

10. የቤተሰብ የወር ገቢ ምን ያህል ነው?-------------

**II. የስነ-ተዋልዶ ጥያ**ቄ**ዎች**

11. ከዚህ እርግዝና በፊት ዘመናዊ የወሊድ መቆጣጠሪያ ትጠቀሚ ነበር?

A. አዎ

B. አልጠቀምም

C. አዎ ግን ከሽፉል

12. ለአስረኛዉ ጥያc አዎ ከሆነ መልስሽ የትኛዉን አይነት?

A. በክንድ ስር የሚቀመጥ

B. በማህፀን ዉስጥ የሚቀመጥ

C. በአፍ የሚዋጥ ኪኒን

D. መርፊ

E. ኮንዶም

F. ሌላ ካለ ተናገሪ-------

13. ለባለፈዉ ልጅሽ ቅድመ ወሊድ ክትትል ነበረሽ?

A. አዎ

B. የለኝም

14. ለ 12ኛዉ ጥያቄ አዎ ከሆነ መልስሠሽ፣ ስንት ጊዜ?

A.አንድ

B.ሁለት

C. ሶስት

D. አራትና ከዚያ በላይ

15. ለባለፈዉ ልጅሽ የእናት ጡት ወተት ብቻዉን ምን ያህል ጊዜ አጠባሽዉ?

A. ከስድስት ወር በታች

B. ሰድስት ወር እና ከዚያ በላይ

16. የባለፈዉ ልጅሽ በጥቅሉ ለምን ያህል ጊዜ ጡት አጠባሽዉ? -----------

17. በቤት ዉስጥ የስነ-ተዋልዶ ጤና አገልግሎት እድትጠቀሚ ማን ነዉ የሚውስነዉ?

A. ራሴ

B. ሁለታችንም/ባልና ሚስት

C. ባሌ ብቻ

18. ጤና ተኮር መረጃዎቺን የምታገኚዉ ከየት ነዉ?

A. ከሚዲያ

B. ከጤና ባለሙያዎች

C. ከጓደኛ/ከዘመድ

D. ምንም መረጃ የለኝም

19. ከአሁኑ እርገዝና በፊት ስንት ልጀች አሉሽ? ------------

20. ወንድ ልጅ አለሽ?

1. አዎ
2. የለኝም

21. የእርግዝናዉ እሳቤ

1. የታሰበ/የታቀደ
2. ያለሰአቱ የተከሰተ
3. ያልተፈለገ
4. ያልተፈለገና ያለሰአቱ የተከሰተ

22. የባለፈዉ እርግዝና ዉጤቱ ምንድነዉ?

A. በህይወት ያለ

B. የሞተ

23. የባለፈዉ ልጅ ፆታዉ ምንድነዉ?

A. ሴት

B. ወንድ

24. የባለፈዉን ልጅ የወለድሽዉ በምን መንገድ ነዉ?

A. በማህጸን

B.በቀዶ ህክምና

25. ከማርገዝሽ በፊት የወር አበባሽ ግዜዉን ጠብቆ ይመጣ ነበር?

A. አዎ

B. አይመጣም

26. ከአሁን በፊት መሃንነት አገጥሞሽ ያዉቃል?

A. አዎ

B. አያዉቅም

27. በባለፈዉ ልጀ እና በአሁኑ እርግዝና መካከል ያለዉ ጊዜ---------------

27.1 የባለፈዉ ልጅሽ የወለድሽበት ቀን-----

27.2 የመጨረሻ የወር አበባሽ ያየሽበት ቀን------
