# Supplementary material for: Prevalence of short interpregnancy interval and its associated factors among pregnant women in Debre Berhan town, Ethiopia
Source: PLoS One. 2021 Aug 2;16(8):e0255613. doi: 10.1371/journal.pone.0255613 (PMC8328324; doi:10.1371/journal.pone.0255613)
Supplement: Attachment — Submitted filename: Rebuttal Letter 2 revised.docx [file pone.0255613.s003.docx]

**Rebuttal Letter**

Dear reviewers and editor,

Thanks for your review and important issues you raised in the paper. Hereunder, kindly get the responses to the points raised.

**Response to editor**

1. I have uploaded the questionnaire (both English version and Amharic version) as Supporting Information.
2. A copy of supporting information file 1 (the dataset of the study) was also uploaded in the system.

**Responses to Reviewer #1**

1. We selected Debre Berhan town because it was the largest town in North Shewa zone where we can find the larger proportion of women compared to other towns of the zone and considering the available budget to conduct the study. (stated in the manuscript)

b) Generally, the culture of Amhara region where Debre Berhan town is located, encourages giving birth of too many children

2. Kebele was defined as “Kebele is the smallest administrative region in Ethiopia approximately comprising 1,000 households.” in the manuscript. The characteristics of the population in all the 9 kebeles is more or less similar. (stated in the manuscript)
3. Relevant information in the study area including access to health care are included in the revised manuscript such as “In the town, there are 29 health institutions: two hospitals (one private and one referral governmental hospital), 3 health centers, 17 drug stores, and 7 private specialty clinics.” And in Ethiopia, the midwives give basic care to all pregnancies and refer those women with serious complications needing urgent referral to hospitals. To eliminate selection bias, the participants were selected randomly from the family folder, and the printed hardcopies were given to the data collectors to access the women and make interview. Besides, being a community-based study, the sampling will not depend on the level of care that the midwives will give.
4. We tried to check for possible interaction of variables; however, we haven’t found significant and/or plausible result.
5. The issue of family planning and interventions were also addressed in the revised manuscript. Actually, it was mentioned not in the background section but in the abstract subsection conclusion and now it’s fixed.
6. Women who had miscarriage/abortion immediately before the current pregnancy were excluded from the study because they are more likely to get pregnant earlier as a result of the pregnancy loss, and they are recommended to have an interval of only six months. Because of the difference in the recommended interval between the pregnancies for those who experienced abortion immediately before the current pregnancy and who didn’t, we excluded them not to overestimate the short interpregnancy interval in a biased manner.

However, those women who were recorded as pregnant in the family folder but had abortion at the study period were not excluded. Despite these, the possibility of bias associated with the exclusion was discussed in the limitation of the manuscript.

Besides, we could not find a true figure of abortion rate and miscarriage rate in Debre Berhan Town as per our search.

Responses to Reviewer #2

ABSTRACT: Under background, the definition of interpregnancy interval (1st sentence) was recast and corrected in the revised manuscript.

INTRODUCTION: Lines 5-7,page 3,paragraph 2 was referenced. Page 3, paragraph 3, lines 14-18 was recast.

METHODS: Concerning “Why was Debre Berham chosen as the study location?” And definition of kebeles, kindly see the responses to reviewer 1 under #1 and #2, respectively.

A more detailed explanation as to how the pregnant women were selected was added in the revised manuscript.

DISCUSSION: Lines 8,page 11,US was written in full 1st. Also RHC (lines 11,page 8.) In page 12, line 8 and line 10 the referencing issue, the authors mix cities and countries in different references was fixed, and the cities were replaced by the respective countries to make it consistent and for better understanding.

Page 12, line 13-14 was recast.

CONCLUSION: “The statement in the last line in the conclusion in page 13, including the non-use of contraceptive as part of the recommendations reduce the incidence of short interpregnancy intervals seems to be at variance with the information in the last paragraph of the discussion before the limitations.” Was addressed in the revised manuscript.

REFERENCES>NO 6- BMJ should be written in full. It is the journal’s actual name, and it’s how they suggest to cite their journal

With kind regards,

The authors
